# When does annual geriatric hip fracture mortality revert to baseline?

**DOI:** 10.3389/fsurg.2024.1359648

**Published:** 2024-10-25

**Authors:** Joseph Bernstein, Alexander Lee, Jaimo Ahn

**Affiliations:** ^1^ Corporal Michael J. Crescenz Department of Veterans Affairs Medical Center, Philadelphia, PA, United States; ^2^ Department of Orthopaedic Surgery, University of Pennsylvania, Philadelphia, PA, United States; ^3^ Department of Orthopaedic Surgery, University of Michigan, Ann Arbor, MI, United States; ^4^ Lieutenant Colonel Charles S. Kettles Department of Veterans Affairs Medical Center, Ann Arbor, MI, United States

**Keywords:** hip fracture, mortality, geriatrics, Veterans, osteoporosis

## Abstract

**Background:**

Geriatric hip fracture patients exhibit high mortality post-injury. It's unclear if and when mortality reverts to baseline. We therefore ask, When, if ever, does the mortality rate of geriatric hip fracture revert to the population-wide baseline rate? How does the mortality rate after geriatric hip fracture compare to the population norms? Understanding this timeline is crucial for assessing disease burden and guiding treatment plans.

**Methods:**

A cohort of 17,868 male patients aged 65–89 years treated for hip fracture within the VA healthcare system was studied. Patients were grouped by age at the time of fracture, and age-specific fractional survival was assessed annually for 10 years. For a comparison control group, a virtual cohort of 17,868 individuals, mirroring the age distribution of the patient group, was created and reduced over 10 cycles according to Social Security Administration expected mortality statistics.

**Results:**

The year-one mortality rate among fracture patients was 35.4%, compared to 6.3% in age-matched controls. By year ten, only 8.5% of the fracture patients remained alive, vs. 39.8% in the general population. The annual risk of dying for patients who survived past the first year was consistently in the range 19%–21% for all subsequent years.

**Conclusion:**

Hip fracture patients who survive the initial injury are still subject to annual mortality risk of approximately 20%, an elevation above population norms persisting for at least a decade. The data underscores the severity of geriatric hip fractures, and suggest that focusing one- or two-year survival rates may not fully capture the severity of the injury.

## Introduction

Hip fractures among geriatric patients are associated with a high risk of death. Studies have consistently shown a mortality rate of at least 20% in the first two years after injury. Despite this, it remains unclear when, if at all, the mortality rate for these patients returns to baseline. Previous studies examining this question have produced inconsistent results, with some reporting a reversion to normal mortality rates after 1 or 2 years (1, 2), while other studies (3, 4) have found a persistently elevated mortality risk.

Understanding when mortality rates for geriatric hip fracture patients return to baseline is crucial for accurately quantifying the population-wide burden of disease. Moreover, this information can help anchor survival prognosis for an individual patient. Long term survival prognosis is clinically important, as, for example, one may want to reserve total hip arthroplasty for femoral neck fracture only for patients likely to live long enough to benefit from that procedure's long-term advantages (5). To our knowledge, there are only few studies examining long-term survival after geriatric hip fracture, and none that reported disaggregated of life expectancy as function of distinct patient age. As such, it is not possible to determine with any specificity if and when the mortality rates of geriatric hip fracture patients of a given age return to their age-specific baseline.

Accordingly, this study aims to contrast the observed mortality rates in male geriatric hip fracture patients with the expected rates in a population without fracture. By doing so, we can determine if and when the mortality rates of geriatric hip fracture return to baseline. The resulting data and analysis not only provide greater theoretical understanding of this condition, but also help orthopaedic surgeons develop means for predicting life expectancy in this vulnerable population to guide treatment accordingly.

## Methods

A cohort of male patients, 65–89 years of age, surgically treated for hip fracture in the Veterans Affairs healthcare system between January, 2000 and December, 2010 was assembled. Median survival of patients within this cohort were previously reported (6). Data were obtained from the VA Informatics and Computing Infrastructure (VINCI) with subjects selected by Current Procedural Terminology (CPT) codes. A total of 17,868 patients were identified.

We recorded each patient's age at surgery and age at death (if applicable). Subsequently, we grouped the patients according to their exact age at the time of fracture, resulting in twenty-five groups ranging from 65 to 89 years of age. We assessed the age-specific fractional survival for each group, annually for the first ten post-operative years.

For a comparison control group, we constructed a *virtual* cohort of 17,868 “normal” male individuals. This control group mirrored the age distribution of the patient cohort. For instance, because our fracture patient data set had 427 patients aged 65 and 726 who were age 75, the same number of subjects of those ages were placed in the virtual cohort. We subjected this virtual cohort to annual attrition for ten cycles, reflecting the expected annual death rate for each age group, using death rate obtained from the Social Security Administration [SSA] (7). That is, each age-band of the virtual cohort was reduced in a given year by the expected death rate in the general population for people of that age. The purpose of this virtual cohort is to demonstrate the difference in survival rates between hip fracture patients and the normal population. This difference represents the excess mortality associated with (and presumably caused by) the fracture.

Although the study used data from existing records and public databases, and thus did not generate any ethical questions *per se*, we adhered to ethical research practices. We ensured that all patient data was anonymized to protect privacy and confidentiality. Approval for the study was likewise granted by the local Institutional Review Board.

## Results

**Figure 1:**
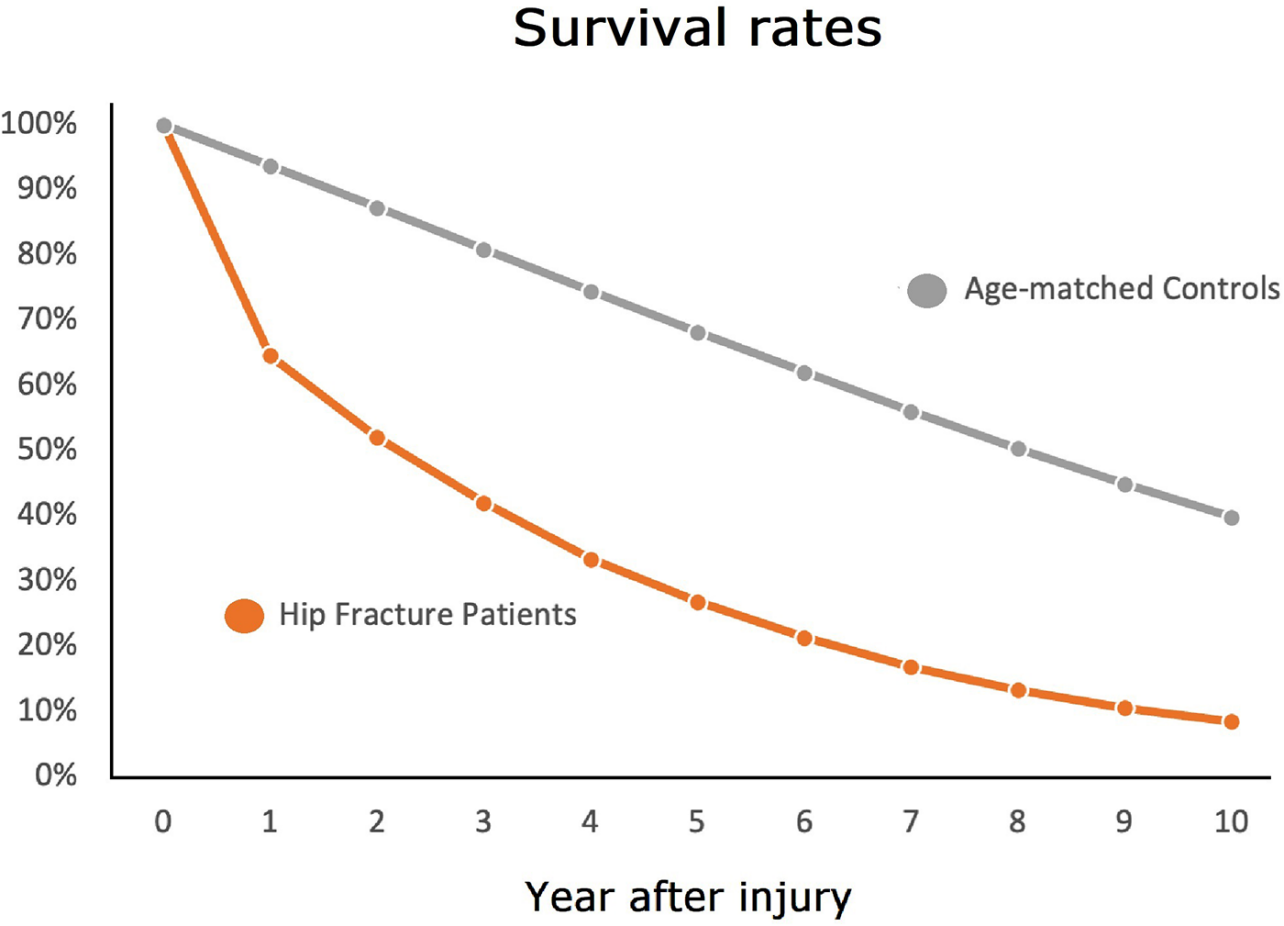
The ten year survival of hip fracture patients (orange line) and that of the general population.

We observed a year-one mortality rate of 35.4% among fracture patients, compared to an anticipated rate of 6.3% in the general population, age-matched cohort. At year ten, only 8.5% of the fracture patients remained alive, whereas the anticipated fractional survival of an age-matched general population cohort would be 39.8% (Figure 1).

**Figure 2:**
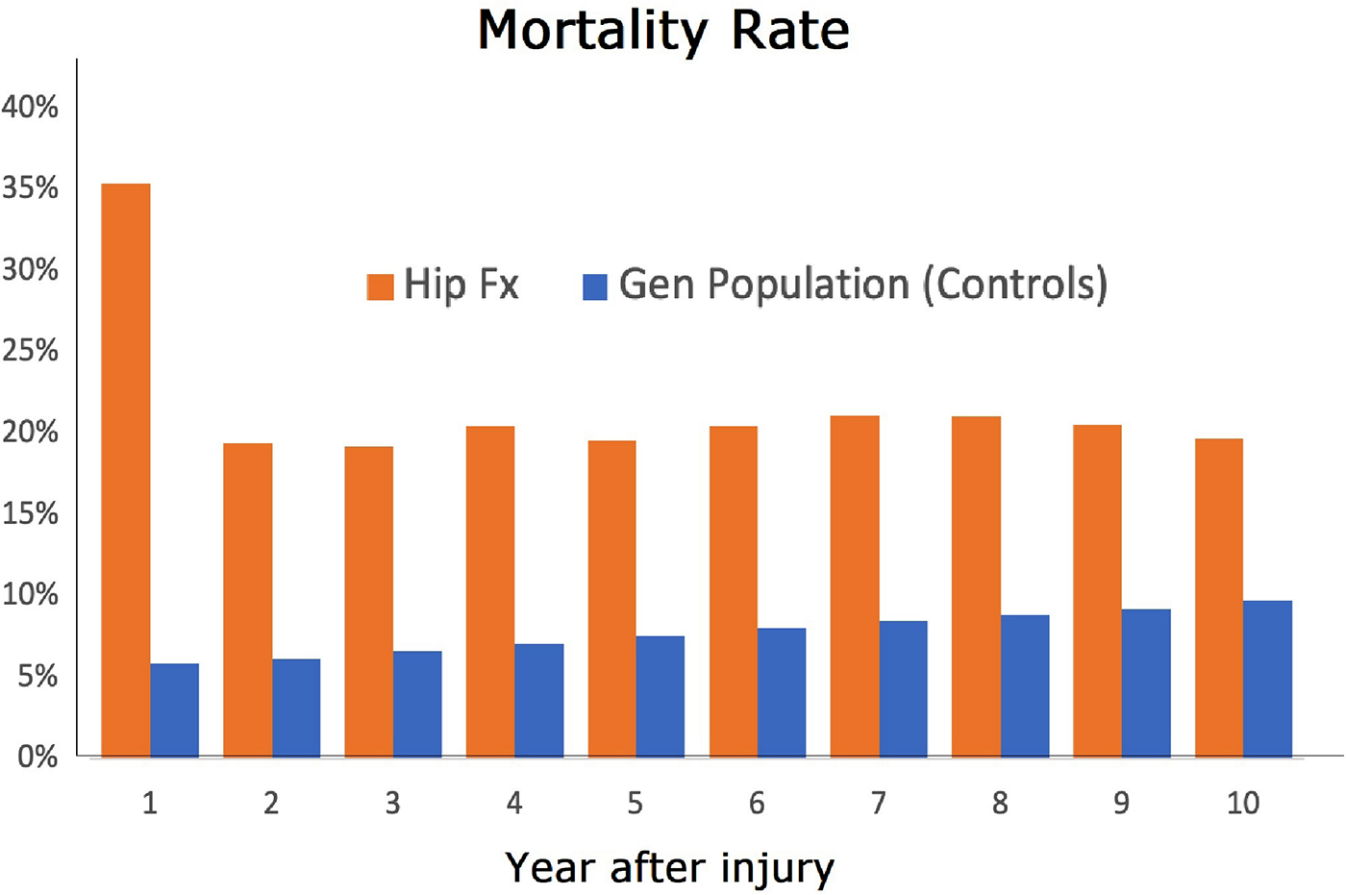
Annual mortality risk by post-fracture year for the fracture cohort (orange bars), compared to the general population (blue bars).

After the first year, the annual risk of dying for all surviving geriatric hip fracture patients was approximately 20%, for all nine other post-fracture years (range: 19%–21%), as shown in Figure 2. The annual fractional survival for both patients and controls of all ages is shown in Table 1.

**Table 1:** Ten year survival as a function of age for geriatric hip fracture contrasted with survival rate of the general population^a^.

	1	2	3	4	5	6	7	8	9	10
65	76% (98%)	66% (97%)	59% (95%)	53% (93%)	48% (91%)	44% (89%)	39% (86%)	33% (84%)	29% (81%)	26% (78%)
66	78% (98%)	68% (96%)	62% (94%)	55% (92%)	48% (90%)	40% (88%)	35% (85%)	30% (83%)	27% (80%)	23% (77%)
67	74% (98%)	64% (96%)	56% (94%)	48% (92%)	41% (89%)	36% (87%)	32% (84%)	27% (81%)	25% (78%)	21% (75%)
68	73% (98%)	63% (96%)	56% (93%)	48% (91%)	42% (88%)	37% (86%)	32% (83%)	27% (80%)	22% (76%)	19% (73%)
69	73% (98%)	63% (95%)	54% (93%)	45% (90%)	38% (87%)	34% (84%)	30% (81%)	26% (78%)	22% (74%)	18% (70%)
70	76% (98%)	65% (95%)	59% (92%)	50% (89%)	42% (86%)	34% (83%)	29% (80%)	25% (76%)	21% (72%)	19% (68%)
71	76% (97%)	64% (95%)	56% (92%)	47% (88%)	40% (85%)	32% (82%)	27% (78%)	22% (74%)	18% (70%)	16% (65%)
72	73% (97%)	59% (94%)	51% (91%)	43% (87%)	37% (84%)	31% (80%)	25% (76%)	22% (72%)	19% (67%)	16% (63%)
73	70% (97%)	58% (94%)	47% (90%)	41% (86%)	35% (82%)	30% (78%)	26% (74%)	22% (69%)	16% (65%)	14% (60%)
74	67% (97%)	57% (93%)	47% (89%)	37% (85%)	32% (81%)	26% (76%)	20% (72%)	16% (67%)	14% (62%)	12% (57%)
75	69% (96%)	55% (92%)	45% (88%)	34% (84%)	29% (79%)	22% (74%)	19% (69%)	14% (64%)	11% (59%)	9% (53%)
76	67% (96%)	55% (91%)	44% (87%)	36% (82%)	29% (77%)	22% (72%)	17% (66%)	14% (61%)	11% (55%)	9% (50%)
77	67% (95%)	56% (91%)	46% (86%)	37% (80%)	29% (75%)	23% (69%)	17% (64%)	14% (58%)	11% (52%)	9% (46%)
78	65% (95%)	50% (90%)	39% (84%)	31% (78%)	24% (73%)	19% (67%)	15% (60%)	12% (54%)	9% (48%)	7% (42%)
79	63% (94%)	51% (89%)	41% (83%)	32% (76%)	26% (70%)	21% (64%)	16% (57%)	13% (51%)	10% (44%)	7% (38%)
80	62% (94%)	50% (88%)	39% (81%)	31% (74%)	25% (67%)	19% (61%)	14% (54%)	11% (47%)	9% (41%)	6% (34%)
81	61% (93%)	49% (86%)	38% (79%)	29% (72%)	22% (65%)	18% (57%)	14% (50%)	10% (43%)	7% (37%)	5% (30%)
82	62% (93%)	48% (85%)	37% (77%)	28% (69%)	22% (61%)	16% (54%)	11% (46%)	7% (39%)	5% (33%)	4% (27%)
83	60% (92%)	46% (83%)	36% (75%)	27% (66%)	20% (58%)	14% (50%)	10% (42%)	7% (35%)	5% (29%)	4% (23%)
84	61% (91%)	47% (82%)	35% (72%)	26% (63%)	19% (55%)	14% (46%)	10% (38%)	7% (31%)	5% (25%)	3% (19%)
85	57% (90%)	43% (80%)	33% (70%)	23% (60%)	18% (51%)	13% (42%)	8% (34%)	5% (27%)	4% (21%)	3% (16%)
86	58% (89%)	47% (78%)	35% (67%)	26% (57%)	18% (47%)	13% (38%)	9% (30%)	6% (24%)	5% (18%)	3% (13%)
87	56% (88%)	41% (75%)	31% (64%)	22% (53%)	15% (43%)	9% (34%)	6% (27%)	3% (20%)	2% (15%)	1% (10%)
88	52% (86%)	37% (73%)	27% (61%)	18% (49%)	14% (39%)	10% (30%)	6% (23%)	3% (17%)	2% (12%)	1% (8%)
89	52% (85%)	39% (70%)	28% (57%)	21% (45%)	14% (35%)	10% (27%)	7% (20%)	5% (14%)	3% (10%)	1% (7%)

^a^ Survival rate of the general population given in parentheses.

## Discussion

Estimating the survival prognosis among geriatric hip fracture patients is a significant responsibility of orthopaedic surgeons providing care. Survival prognosis can help estimate burden of disease. Survival prognosis can also help guide the treatment of individual patients and offer salient information to patients and their families.

In the realm of survival prognosis, we have previously reported the median survival of geriatric hip fracture patients as function of age (6). Median survival can help anchor a Bayesian process for estimating a particular patient's expected survival, but it is not enough. Reference to the general population over an extended time horizon, as we have done here, can be additionally informative.

In this study we compared the rates of survival in a large cohort of older male hip fracture patients to the survival expected in the general population. We have demonstrated that the rate of survival among geriatric hip fracture patients is markedly and persistently lower than as compared to the general population. Accordingly, the answer to the question, “when does annual geriatric hip fracture mortality revert to baseline?” is that it does not– at least not within the first 10 years after fracture. As shown in the table, the relative risk of a fracture patient dying in year three is triple that of the age-matched general population and still nearly double in year 10. Likewise, the 5 year survival of geriatric hip fracture patients for all ages younger than 84 is lower than that of people in the general population who are ten years older.

This study has several recognized strengths and weaknesses. Notably, our research encompasses a vast number of patients, a data set uniquely large enough to provide detailed information for each year of age. This method contrasts with other studies that assign patients to five-year age range bins, 65–69, 70–74, and so on (8). Use of ranges can introduce imprecision. For instance, were we to calculate a grouped five-year survival for the cohort aged 65–69, the resulting 43% figure would not be reflective of any true data point within that range (where survival rates at ages 65–69 were 48%, 48%, 41%, 42%, and 38%, respectively). This grouped figure of 43% indeed deviates by more than 10% (5 percentage points) from both the highest and lowest survival rates within the age group.

Another strength of this study lies in the use of the VINCI system, which fully accounts for all patients. As a result, we were able to report on complete survival rates, rather than compensating for censored data through methods such as the Kaplan-Meier estimator. Additionally, we compared our patients to population norms, avoiding the potential pitfalls associated with model-matching patients to controls believed to be comparable in all aspects except for the presence of a fracture.

The major limitation of the study is that it reported on males only. Although geriatric hip fracture in males is a clinically significant problem, with tens of thousands of such cases annually in the United States alone, the injury is more commonly seen among females (9). Accordingly, the findings reported here may not generalize to prototypical geriatric hip fracture patient. This potential problem is inherent in studies reporting VA data exclusively, as the VA patient population is overwhelmingly male.

Additionally, it is recognized that VA patients may differ from their age-matched civilian counterparts in terms of co-morbidities and socio-economic factors (10). Also, the quality of care within the VA system may also vary from that provided in civilian hospitals (11). However, the extent and impact of these differences remain unknown. We are further unable to present data based on race as this information was not recorded for approximately 25% of the patients. Lastly, the lack of patient-specific features (such as medical history and laboratory values) in our data set precludes any comment regarding the relationship between pre-injury frailty (12) and life expectancy.

It must be reiterated that our study addresses a population-based question: When, in general, do the mortality rates of geriatric hip fracture patients of a given age return to their age-specific baseline? That is, this is a question about the epidemiological behavior of a condition. It is not a question that directly addresses the prognosis of a specific individual. While geriatric hip fracture is indeed associated with an increased risk of mortality (as our top-line result reiterates), there are many patients within our study group who survived for durations longer than the average would suggest. Therefore, it is essential that the life expectancy prognosis of each patient be considered individually and not forced into the Procrustean bed of population averages. This approach is consistent with the medical maxim, “medicine is practiced not on mankind in general, but on every individual in particular.”

In sum, our study demonstrates that geriatric hip fractures are associated with an elevated risk of mortality for at least a decade following the injury. We found a consistent 20% annual mortality risk from the second through to the ninth year after injury, highlighting the life-threatening implications of geriatric hip fractures beyond perioperative complications. Our findings suggest, moreover, that studies focusing only on survival rates within the first two years post-injury may not fully capture the severity of the injury.

## Data Availability

The data analyzed in this study is subject to the following licenses/restrictions: The study was based on data provided by The Department of Veterans Affairs within the VINCI system. Requests to access these datasets should be directed to joseph.bernstein@va.gov.
